# Where Do Patients Go for Treatment of Urethritis?

**DOI:** 10.5812/numonthly.16993

**Published:** 2014-05-15

**Authors:** Mustafa Burak Hoscan, Ahmet Tunckiran, Taylan Oksay, Alper Ozorak, Hakan Ozkardes

**Affiliations:** 1 Alanya Practice and Research Center, Department of Urology, Faculty of Medicine, Baskent University, Alanya, Turkey; 2 Department of Urology, Faculty of Medicine, Suleyman Demirel University, Isparta, Turkey; 3 Department of Urology, Faculty of Medicine, Baskent University, Ankara, Turkey

**Keywords:** Patient Acceptance of Health Care, Therapeutics, Urethritis

## Abstract

**Background:**

Urethritis is characterized by urethral inflammation, and it can result from both infectious and noninfectious conditions. Physicians and other health care providers play a critical role in preventing and treating urethritis.

**Objectives:**

The aim of this study was to describe and identify predictors of health care seeking behavior among men with urethritis.

**Patients and Methods:**

In total, 98 male patients aged between 16 to 52 years-of-age (mean 30.9 ± 8.0 years), who attended our clinic with symptoms of urethritis, were included in the study. We conducted face-to-face interviews with the patients using a 9-item survey questionnaire. Patients were divided into three groups according to their level of education as follows: group I (n = 44), elementary school; group II (n = 38), high school; and group III (n = 16), university.

**Results:**

Among the 98 patients evaluated, the source of treatment was physicians in 44 patients (44.9%), drugstores in 38 cases (38.77%), and friends in 16 patients (16.32%). There was a statistically significant difference found between the groups according to the source of treatment (P < 0.001). The most common factors associated with seeking care from other sources, rather than physicians, were economic reasons in 19 patients (35.18%), confidentiality concerns in 24 (44.4%), and ease of access in 11 patients (20.37%).

**Conclusions:**

A substantial proportion of patients with urethritis sought help from other sources, rather than physicians. The results of our study show that the patients with higher levels of education were more likely to seek help from health care services. It is important to promote the public’s knowledge through informative studies and educational materials in order to encourage patients to seek rapid and effective treatment from proper sources.

## 1. Background

Urethritis is characterized by urethral inflammation, which can result from infectious and noninfectious conditions (1). Even though there have been significant advances in the prevention, diagnosis, and treatment of urethritis, the occurrence of this condition continues to rise. Physicians and other health care providers play a critical role in the prevention and treatment of urethritis. If this problem is left untreated or treated inappropriately, urethritis may cause orchitis, epididymitis, and prostatitis (1). Urethritis commonly affects men during their reproductive years (2). Treatment should be initiated as soon as possible after a diagnosis has been made, because incomplete treatment or delays in therapy in this group of patients increases the likelihood of complications and transmission of the infection to others (3). Although the possibility of transmission by symptomatic infected patients to their partners is clearly unknown, several studies have shown that up to one third of patients with symptomatic urethritis continue sexual activity in the interval between the onset of symptoms and their presentation for diagnosis and therapy (4, 5). Therefore, developing a better understanding of health care seeking behaviors of patients with urethritis might provide important information which can assist in the development of new interventions and preventive measures.

## 2. Objectives

We aimed to present the results of a survey to describe and identify predictors of health care seeking behavior among men with urethritis.

## 3. Patients and Methods

We included 98 male patients between the age of 16 to 52 years (mean 30.9 ± 8.0 years) who attended our clinic with symptoms and diagnosis of urethritis. The patients were interviewed face to face using a 9-item survey questionnaire. The patients were asked to fill in a questionnaire that was administered by a physician. This questionnaire was conducted on a one-on-one basis in a private counseling room. The voluntary nature of the study was stressed and informed consent was obtained from each patient. Basic demographic data was obtained along with information concerning the disease, possible source of infection, location of treatment, and reason for choosing the advised treatment. According to their education level, patients were divided into three groups as follows:

group I (n = 44), elementary school;
group II (n = 38), high school; and
group III (n = 16), university education.

Statistical analyses were performed using SPSS software for Windows (version 11.0, SPSS Inc. Chicago, IL, USA). The differences among the groups in terms of age were evaluated by a one-way ANOVA. A Kruskal-Wallis test was performed to assess the differences between the groups’ education levels and for intergroup comparison. This was followed by pair wise comparison of groups using a Mann-Whitney U test. Differences were considered significant when P < 0.05.

## 4. Results

**Figure 1.**
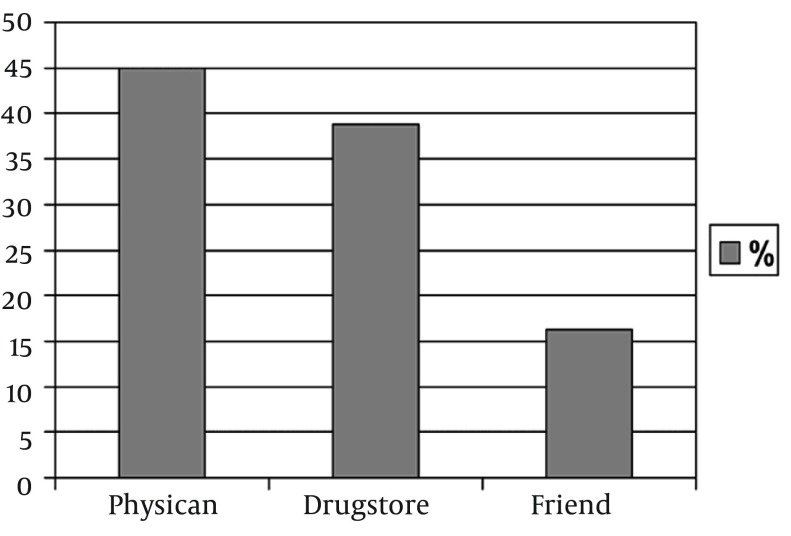
Source of Treatment in Patients With Urethritis

**Figure 2.**
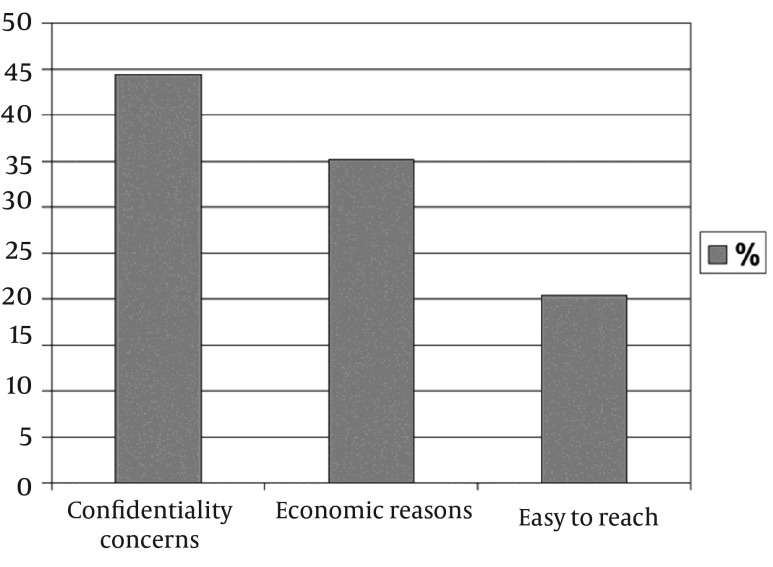
Factors Associated With Seeking Care at Sources Rather Than Medical Services

The mean age was 32.09 years (range, 19-50 years, SD = 8.9), 29.74 years (range, 20-52 years, SD = 7.5), and 30.38 years (range, 16-42 years, SD = 6.8) in group I, group II, and group III, respectively. There was no statistically significant difference found with regard to mean age among the groups (P = 0.404). Among the study’s 98 patients, the sources of treatment in 44 cases (44.9%) were physicians, drugstores in 38 (38.77%), and friends in 16 patients (16.32%) (Figure 1). All patients had a history of unprotected sexual intercourse. The given or advised therapies from the various sources, other than the physicians, were not standard therapy regimens and were frequently insufficient or overtreated. The most common factors associated with seeking care at sources other than physicians were; economic reasons in 19 patients (35.18%), confidentiality concerns in 24 (44.4%), and ease of access in 11 patients (20.37%) (Figure 2).

There was a statistically significant difference among the groups with respect to the source of treatment (P < 0.001). When the groups are compared, there was a statistically significant difference found between groups I and II (P < 0.001), and between groups I and III (P = 0.008). The difference between groups II and III was not statistically significant (P = 0.170). The results of our study showed that the patients with higher levels of education were more likely to seek help from health care services.

## 5. Discussion

The preferred medical management of persons with urethritis is to visit a health care clinic for diagnostic testing, treatment, and counseling (6). Effective treatment of urethritis depends on infected persons being able to recognize symptoms early, seek and receive prompt treatment with effective drugs, and successfully refer their partners (7). A lack of successful treatment increases the duration of the infection, thus contributing to complications and transmission of urethritis (8). Efforts must be made to promote more timely care-seeking behavior in these cases by educating the public about the adverse effects and possible complications of untreated urethritis, as well as by increasing the overall quality of the services that they provide (7).

In urethritis treatment, there are increasing concerns about the widespread use as well as misuse of antibiotics in terms of the development of microbial resistance, adverse reactions, and changes in microbial flora (6). It has also been reported that in several countries, in spite of regulations, the majority of people still seek care for urethritis from drugstores (9-12). In our study, the main source of treatment was drugstores in 38.77% of patients, and the service that people receive is often of poorer quality than those under medical supervision (10). Furthermore, it has been well established that the primary goal of business is profit, rather than patient’s wellbeing (13). In a study by Chalker et al. it was reported that even though 74% of pharmacists and drug sellers knew that they should not treat sexually transmitted diseases (STD), in reality, 84% provided treatments for them (14). Moreover, when these patients were treated, they had not received the correct combination of drugs for treatment using syndromic management, as only 12% had been given tetracycline or doxycycline, and the medication had not been given for a sufficient duration (14). In another study, it was reported that 82% of patients were treated in drugstores and only 1.5% of the regimens prescribed conformed to national guidelines. In our study, the given or advised therapies from sources other than physicians were also not standard therapy regimens and were commonly insufficient or overtreated (12).

In the 1990s, self-medication for the treatment of urethritis became increasingly common among a wide range of patients, in addition, patients used alternative therapies without their physician’s knowledge (14-16). Following these findings, the World Health Organization’s (WHO) consultative group (17) reported that the role of the pharmacist has been changing over the past two decades, with self-treatment increasing worldwide. They also reported that pharmacists were responsible for recommending safe and efficacious medicine, furthermore, sound advice was essential and the pharmacist was required to be a responsible communicator, supervisor, and health promoter (17). In fact, as we previously mentioned, their actual behavior was found to be poor. In one study, it was reported that questionnaire results among pharmacists suggested a lack of knowledge and that their level of advice was dramatically lacking, both in the questionnaire and in practice (14). Even though both the quality of drug treatment and advice were unacceptably poor (14), people with urethritis went to private pharmacists because it was cheaper than a physicians visit and the service was more accessible and less controlled (18).

The findings of one study indicated that clinics were used by a substantial minority of patients with urethritis when compared to the proportion of the population attending clinics (19). Medical services have been described as having several benefits (20). The reported benefits of using medical services include; the patient's history was taken, patients were examined, laboratory tests were carried out, treatment was more effective, especially when injections were prescribed, and the patients were reviewed after treatment. However, a number of limitations inhibiting the use of medical services were also reported, such as; requests to bring sexual partners, long waiting queues, appointment delays, shortage of drugs, lack of privacy, high cost, and negative attitudes of the staff (20).

Benjarattanaporn et al. reported that 39% of men presenting with urethritis were initially treated at drugstores, 29% at private clinics, and 19% at government clinics. Failure to respond to therapy was the primary reason for seeking additional care. In addition, men with an STD reported that convenience, affordability, and lack of embarrassment were associated with their choice of treatment site (10). Another study concerning the location where people received treatment for a STD reported that private practice (49%) was the most frequently mentioned place to which participants had sought treatment (2). Among the remaining patients, 8% had gone to an “other clinic,” 7% to an emergency room, 5% to a STD clinic, 5% to a family planning clinic; 23% did not mention any previous place where they had been treated for their STD, while 3% mentioned multiple sites. A study analyzing the factors associated with the choice of STD health care facility demonstrated the importance of having medical services close to where the patient lived or worked (21). This study also reported that affordability and confidentiality were associated with the choice of STD treatment facility among male patients.

In a study of STD patients, the participants were asked to identify up to three reasons why they had selected that particular STD clinic on the day of the interview (22). The most common reasons included; availability of walk-in services or same-day appointments (68%), lower cost of care (59%), privacy or confidentiality concerns (43%), convenience of the STD clinic's location (40%), and expert care (34%). Another study from the Netherlands reported that unprofessional attitudes and embarrassment were reasons for not seeking STD services from their regular provider (23). A study to assess the health-seeking behavior of STD patients demonstrated that the choice of a particular health facility for general health care was determined mainly by convenience of the location (56%), privacy (18%), and affordability (12%), of the services. The most common factors associated with seeking care at sources other than physicians in our study were; economic reasons (35.18%), confidentiality concerns in (44.4%), and ease of access (20.37%).

A substantial proportion of patients with urethritis sought help from sources other than physicians. Uncompleted treatment or delay in therapy in this group of patients increases the likelihood of complications and transmission of infection to others. The results of our study showed that the patients with higher level of education were more likely to seek help from health care services. It is important to promote knowledge of this condition and to educate the public with informative studies and educational materials in order to encourage patients to attend clinics for rapid and complete treatment.
